# Supplementary material for: A Critical View of the Application of the APEX Software (Aqueous Photochemistry of Environmentally-Occurring Xenobiotics) to Predict Photoreaction Kinetics in Surface Freshwaters
Source: Molecules. 2019 Dec 18;25(1):9. doi: 10.3390/molecules25010009 (PMC7017383; doi:10.3390/molecules25010009)
Supplement: Supplementary file 1 [file molecules-25-00009-s001.zip › 3.2.4_gcc-4.4.0.zip!/3.2.4_gcc-4.4.0/3.2.4_gcc-4.4.0/doc/octave/html/liboctave/Ordinary-Differential-Equations.html]

Ordinary Differential Equations - Octave C++ Classes


Next: Differential Algebraic Equations,
Previous: Quadrature,
Up: Top


---

## 11 Ordinary Differential Equations

— : **ODE\_options** (void)  
— : **ODE\_options** (const ODE\_options &opt)

— : ODE\_options& **operator =** (const ODE\_options &opt)

— : void **init** (void)

— : void **copy** (const ODE\_options &opt)

— : void **set\_default\_options** (void)

— : void **set\_absolute\_tolerance** (double val)

— : void **set\_initial\_step\_size** (double val)

— : void **set\_maximum\_step\_size** (double val)

— : void **set\_minimum\_step\_size** (double val)

— : void **set\_relative\_tolerance** (double val)

— : double **absolute\_tolerance** (void)  
— : double **initial\_step\_size** (void)  
— : double **maximum\_step\_size** (void)  
— : double **minimum\_step\_size** (void)  
— : double **relative\_tolerance** (void)

— : **ODE** (void)  
— : **ODE** (int n)  
— : **ODE** (const ColumnVector &state, double time, const ODEFunc &f)

— : virtual int **size** (void) const

— : virtual ColumnVector **state** (void) const

— : virtual double **time** (void) const

— : virtual void **force\_restart** (void)

— : virtual void **initialize** (const ColumnVector &x, double t)

— : virtual void **set\_stop\_time** (double t)

— : virtual void **clear\_stop\_time** (void)

— : virtual ColumnVector **integrate** (double t)

— : void **integrate** (int nsteps, double tstep, ostream &s)

— : Matrix **integrate** (const ColumnVector &tout)  
— : Matrix **integrate** (const ColumnVector &tout, const ColumnVector &tcrit)
